# Infernape uncovers cell type–specific and spatially resolved alternative polyadenylation in the brain

**DOI:** 10.1101/gr.277864.123

**Published:** 2023-10

**Authors:** Bowei Kang, Yalan Yang, Kaining Hu, Xiangbin Ruan, Yi-Lin Liu, Pinky Lee, Jasper Lee, Jingshu Wang, Xiaochang Zhang

**Affiliations:** 1 Department of Human Genetics, The University of Chicago, Chicago, Illinois 60637, USA;; 2 Department of Statistics, The University of Chicago, Chicago, Illinois 60637, USA;; 3 The Neuroscience Institute, The University of Chicago, Chicago, Illinois 60637, USA

## Abstract

Differential polyadenylation sites (PAs) critically regulate gene expression, but their cell type–specific usage and spatial distribution in the brain have not been systematically characterized. Here, we present Infernape, which infers and quantifies PA usage from single-cell and spatial transcriptomic data and show its application in the mouse brain. Infernape uncovers alternative intronic PAs and 3′-UTR lengthening during cortical neurogenesis. Progenitor–neuron comparisons in the excitatory and inhibitory neuron lineages show overlapping PA changes in embryonic brains, suggesting that the neural proliferation–differentiation axis plays a prominent role. In the adult mouse brain, we uncover cell type–specific PAs and visualize such events using spatial transcriptomic data. Over two dozen neurodevelopmental disorder–associated genes such as *Csnk2a1* and *Mecp2* show differential PAs during brain development. This study presents Infernape to identify PAs from scRNA-seq and spatial data, and highlights the role of alternative PAs in neuronal gene regulation.

For ∼70% of human genes, differential polyadenylation alters the 3′ untranslated regions (3′ UTRs) that may regulate mRNA metabolism and protein expression (Derti et al. 2012; Tian and Manley 2017). Cleavage and polyadenylation (C/P) are regulated by *cis*-acting RNA sequences and their interaction with *trans*-acting C/P protein complexes (Shi 2012; Elkon et al. 2013; Gruber and Zavolan 2019; Mitschka and Mayr 2022). The cleavage and polyadenylation site (PA) is often a CA dinucleotide defined by surrounding sequence motifs such as the polyadenylation signal (PAS) (Tian and Manley 2017; Yoon and Shi 2022). Differential PAs critically regulate neural development and synaptic plasticity in mice (An et al. 2008; Bae et al. 2020), and human mutations in a core C/P protein CSTF2 or the modulatory cleavage factor I (CFI) complex cause neurodevelopmental disorders (Gennarino et al. 2015; Grozdanov et al. 2020; de Prisco et al. 2023). Alternative polyadenylation (APA) alters the length of 3′ UTRs, whereas alternative splicing may lead to alternative last exons (ALEs) and intronic polyadenylation sites (IPAs). ALE–IPA has been shown to alter neuronal mRNA localization (Taliaferro et al. 2016) and polarize neuronal functions (Yap et al. 2016). Increasing evidence has linked variations in PA usage to human trait-associated genetic loci (Mittleman et al. 2020; Li et al. 2021b). Thus, PAs are important for the regulation of gene expression and variations of organismal phenotypes.

Developmental expansion of the neocortex is unique in mammals: in the mouse dorsal forebrain, radial glial progenitors (RGCs) start to generate cortical neurons and intermediate progenitor cells (IPCs) at embryonic day (E) 11.5, and layers of excitatory neurons are sequentially born by E18.5 (Götz and Huttner 2005; Lui et al. 2011; Geschwind and Rakic 2013; Greig et al. 2013; Bae et al. 2015; Hevner 2019). Inhibitory neurons are largely generated in the ganglionic eminence and migrate tangentially into the neocortex (Marín and Rubenstein 2001). Shorter 3′ UTRs are observed in proliferating cells and early embryonic development, whereas adult neural tissues tend to express distal PAs (Sandberg et al. 2008; Ji et al. 2009; Mayr and Bartel 2009; Miura et al. 2013). The expression of polyadenylation factors and RNA-binding proteins (RBPs) such as *Elavl3/4* has been reported to affect PA usage (Ince-Dunn et al. 2012; Gruber and Zavolan 2019). Despite the importance of PA regulation in neural development and disorders, its expression and spatial distribution across brain cell types remain to be fully understood.

Single-cell RNA-seq (scRNA-seq) and spatial transcriptomic methods based on oligo(dT) priming and barcoding have revolutionized our understanding of cellular heterogeneity in animal tissues (Klein and Macosko 2017). Recent studies have uncovered more than 100 neuronal subtypes that are transcriptionally specified in the mouse brain (Saunders et al. 2018; Tasic et al. 2018; Zeisel et al. 2018; Cao et al. 2019; Rodriques et al. 2019; Di Bella et al. 2021; Ruan et al. 2021; Zhang et al. 2021; Chen et al. 2022). Oligo(dT)-captured scRNA-seq reads align near cleavage sites and provide information to evaluate cell type–specific PAs. Several analytical methods have been developed to identify and/or quantify PAs from mammalian scRNA-seq data such as scAPA (Shulman and Elkon 2019), Sierra (Patrick et al. 2020), scDAPA (Ye et al. 2020), MAAPER (Li et al. 2021c), SAPAS (Yang et al. 2021), scAPAtrap (Wu et al. 2021), SCAPTURE (Li et al. 2021a), scDaPars (Gao et al. 2021), and SCAPE (Zhou et al. 2022). These recent studies uncovered PA usage in mouse immune and developing brain cells, single nuclei from the early embryos (Agarwal et al. 2021), major GABAergic neuron types (Yang et al. 2021), and a large collection of samples (Zhu et al. 2022), suggesting that scRNA-seq is suitable for identifying cell type–specific PAs. However, it remains challenging to call differential PAs reliably owing to technical biases associated with scRNA-seq library preparation. Moreover, oligo(dT) capture-based spatial transcriptomics has been increasingly used to study transcription levels (Rodriques et al. 2019; Chen et al. 2022; Moses and Pachter 2022), but the spatial distributions of transcriptome-wide PA usages remain unexplored.

Here we seek to uncover cell type–specific PAs in the mouse brain using scRNA-seq and spatial transcriptomic data. We have developed Infernape, an analytical pipeline that integrates reference PAs with de novo inference to identify cell type–specific APA and IPA events. We have benchmarked Infernape with concurrent single-cell PA methods and shown its application in calling differential PAs during cortical neurogenesis. We further applied Infernape to the adult brain and uncovered differential PAs using single-cell and spatial transcriptomic information. Our results nominate cell class–specific and cell type–specific PAs in the mouse brain.

## Results

### Infernape identifies cell type–specific PAs from scRNA-seq data

**Figure 1.**
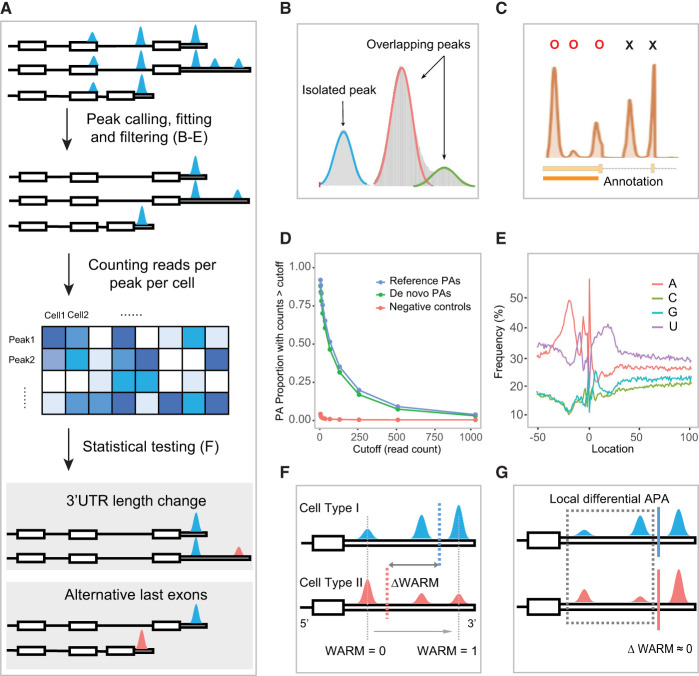
Schematic overview of the Infernape pipeline. (*A*) Overview of the Infernape workflow. (*B*) Overlapped peaks are fitted and separated with a Gaussian mixture model. Peak mode detection and filtering. (*C*) Peak modes are first identified based on smoothed unique UMI coverage curves, and non-3′-UTR region peaks are filtered out. Using *Ephb2* (E18.5 mouse brain) as an example, peak modes in reference 3′-UTR regions are labeled by Os, and excluded peak modes are labeled by Xs. (*D*) The proportion of Infernape-identified peaks associated with de novo PAs, known PAs, and corresponding read counts in a 150-bp window downstream from each peak mode. The read counts were derived from bulk RNA-seq of the E14.5 RGCs and neurons. PAs of genes that are not expressed in the scRNA-seq data are used as the negative control. The read counts of de novo PAs (green) are comparable to that of known PAs (blue), and both are substantially higher than the negative control (red). (*E*) Nucleotide frequency around PAs identified by Infernape (adult mouse brain data) showing the A- and U-rich regions. Position 0 indicates the cleavage/PA site. (*F*) An illustration showing the weighted average relative mode (WARM) value, which summarizes the relative PA usage of a gene based on all its PAs. Blue and red dashed lines indicate the WARM value for the two cell types. Gray dashed lines indicate the lower and upper bound for the WARM value. (*G*) An illustration showing the maximum difference in proportion change (MPRO) value, which measures the greatest local PA change. Blue and red lines indicate similar WARM values for the two cell types for which the local difference may be masked by the averaging effect. MPRO results indicate that the two proximal PAs show significant local differential usage.

**Figure 2.**
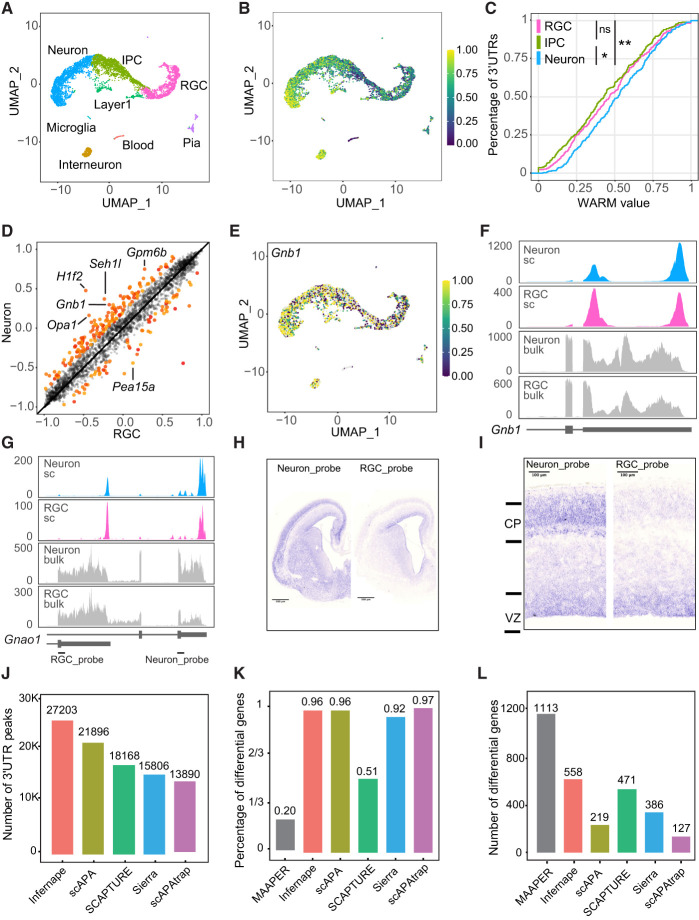
Dynamic PAs during cortical neurogenesis and benchmarking of Infernape. (*A*) UMAP showing main cell types in the E14.5 dorsal mouse forebrain (5482 single cells). Data were analyzed from a previous study (La Manno et al. 2021). (*B*) UMAP showing transcriptome-wide average WARM values across cells. Higher WARM values indicate higher usage of distal PAs. (*C*) Empirical cumulative density curves of average WARM values for neuron, RGC, and IPC. Cell type–specific WARM values are calculated for APA signals of all multiple-PA genes. Wilcoxon rank-sum tests were used to test the overall APA difference between cell types. (*) adj.*P*-value < 0.05, (**) adj.*P*-value < 0.01, (ns) nonsignificant. (*D*) Scatter plot of WARM values showing 3′-UTR lengthening in neurons. Each dot represents a transcript, and the *x*/*y*-axes represent WARM values for the two cell types in comparison. Significant and nonsignificant differential APA events are colored red and gray, respectively. (*E*) UMAP showing WARM values for the *Gnb1*:ENSMUST00000165335.7 transcript were higher in neurons than in RGCs. (*F*) Coverage plot of the *Gnb1*:ENSMUST00000165335.7 transcript for neurons and RGCs, from both scRNA-seq (blue and pink) and bulk RNA-seq data (gray). (*G*) Coverage plot of the *Gnao1* gene for single-cell and bulk RNA-seq of neurons and RGCs. (*H*) RNA in situ hybridization (ISH) results of *Gnao1* in the E15.5 mouse brain (coronal section). (*I*) RNA ISH results of *Gnao1* (zoom-in for *H*): The distal PA (Neuron_probe) shows a higher signal in the cortical plate (CP) than in the ventricular zone (VZ), whereas the proximal PA (RGC_probe) shows a higher signal in the VZ than in the CP. (*J*) The number of peaks identified by different PA methods using the E14.5 scRNA-seq data. MAAPER was not included here because it outputs PA coordinates instead of peak coordinates. (*K*) The proportion of differential PA genes that is identified by at least one of the other single-cell PA methods. (*L*) The number of differential PA genes that are shared by at least one of the other single-cell PA methods.

We have developed the inferring alternative polyadenylation from scRNA-seq (Infernape) pipeline to investigate PA usage from single-cell and spatial transcriptomic data generated from the 10x Genomics platforms. In conjunction with cell-type identification (Zheng et al. 2017; Butler et al. 2018), Infernape performs PA inference and quantification through a multistep process including stringent peak calling, fitting, filtering, and statistical testing (Methods) (Fig. 1A). Briefly, Infernape aims to achieve accuracy in assigning read counts to the corresponding cleavage sites by (1) leveraging Gaussian mixture models to separate overlapping peaks (Fig. 1B), (2) determining the interval of peak-to-PA distance using single-PA–single-peak genes (Supplemental Fig. S1A), (3) using integrated PA references (Wang et al. 2018; Herrmann et al. 2020; Agarwal et al. 2021) to annotate the identified peaks (Fig. 1C), and (4) inferring de novo PAs based on called peaks and their proximity to PAS sequences (Methods) (Fig. 1D,E; Supplemental Table S1). Finally, Infernape incorporates a Dirichlet-multinomial test to assess differential polyadenylation across cell types, allowing for an unrestricted number of peaks (PAs) for any given gene (Methods). Infernape mitigates excessive false positives in PA identification and the detection of differential PA events.

We introduce two measures to quantify PA usage. We propose the weighted average relative mode (WARM) value to summarize the proximal-distal relative PA usage of a gene based on all its PAs for either within-UTR APA or across-UTR IPA events (Methods) (Fig. 1F). To further capture local differential PA patterns and provide an intuitive measure of proportional PA changes, we introduce maximum difference in proportion change (MPRO). MPRO ranks differential PA events by contrasting all possible peak pairs across cell types/conditions under a difference-in-difference scheme (Methods) (Fig. 1G). WARM and MPRO together provide a comprehensive measure to quantify and rank differential PA events (Supplemental Fig. S1B).

We simultaneously consider effect size, statistical significance after multiple testing adjustments, and the corresponding peaks’ expression levels to determine differential PA events. Specifically, the following thresholds were used: (1) the absolute MPRO is >20%; (2) the Benjamini–Hochberg adjusted *P*-value is not greater than 0.05; and (3) the PA signal is detected in ≥5% cells for each cell group in comparison. The Infernape package is available on GitHub, and we have developed a web-based portal to show differential PA test results across cell types in the developing mouse brain.

### Dynamic PA during cortical neurogenesis

To benchmark Infernape and understand cell type–specific PA patterns in cortical development, we reanalyzed scRNA-seq data of the E14.5 mouse dorsal cortex (La Manno et al. 2021), representing the peak of cortical neurogenesis. We analyzed 5482 single cells and identified main cell types including RGC, IPC, and neurons (Fig. 2A; Supplemental Fig. S2A). Infernape uncovered 24,765 peaks associated with annotated PAs and 2439 with de novo PAS motifs (Supplemental Table S1). Infernape detected differential PAs in 581 genes (|MPRO| > 20%, adj.*P*-value < 0.05), and the differential PA events were not significantly affected by peak calling parameters (Supplemental Fig. S1C). Based on the average WARM values for all multipeak/PA genes per cell, the average length of 3′-UTR usage was longer in neurons (Fig. 2B,C), and more genes showed longer 3′ UTRs in neurons than in RGCs (143 vs. 61) (Fig. 2D; Supplemental Table S2). These results indicate that Infernape detected 3′-UTR lengthening during neuronal differentiation from scRNA-seq data.

To cross-validate Infernape findings, we compared APA and IPA events from E14.5 scRNA-seq data to bulk RNA-seq results of isolated cell types. Briefly, we used flow cytometry to enrich *Eomes:EGFP*-negative RGCs (Zhang et al. 2016) and *Tubb3:EGFP*-positive neurons (Yang et al. 2023) from the E14.5 mouse dorsal forebrains and sequenced polyadenylated RNA. First of all, the 2439 peaks with de novo PAS motifs were supported by comparable bulk RNA-seq read counts to peaks with annotated PAs (Fig. 1D). We identified PA changes between bulk E14.5 RGCs and neurons using REPAC (Imada et al. 2023), and the results validated 104 out of 581 differential PA genes identified by Infernape. Although it remains challenging to identify PA changes from bulk RNA-seq (Shah et al. 2021), these observations suggest that the 3′-end-based scRNA-seq uncovers overlapping PA events from bulk samples. For instance, the *Gnb1* gene expresses longer 3′ UTRs in neurons than in RGCs in both scRNA and bulk RNA results (Fig. 2D–F). In parallel to APA, Infernape reported IPAs between cell types (Supplemental Fig. S2B; Supplemental Table S2): There were 37 genes showing an enriched distal last exon in RGCs such as *Klc1* (Supplemental Fig. S2C,D), and there were 39 genes showing higher distal last exon usage in neurons such as *Gnao1* (Fig. 2G). We performed RNA in situ hybridization with probes against the *Gnao1* RGC and neuron 3′ UTRs and validated their enriched expression in the E15.5 ventricular zone (VZ; enriched for RGCs) and the cortical plate (CP; enriched for neurons), respectively (Fig. 2H,I). These results indicate that a fraction of Infernape-identified PAs were cross-validated by bulk RNA-seq and wet experiments.

### Benchmarking Infernape

We compared Infernape to concurrent single-cell PA methods such as scAPA (Shulman and Elkon 2019), Sierra (Patrick et al. 2020), SCAPTURE (Li et al. 2021a), scAPAtrap (Wu et al. 2021), and MAAPER (Li et al. 2021c) by applying them to the same E14.5 scRNA-seq data set. First, Infernape identified more peaks than the other tested methods, likely owing to the peak fitting and filtering processes (Methods) (Fig. 2J). When focusing on differential PA genes between RGCs and neurons, all the methods detected hundreds of differential PA events, except MAAPER, which identified about one magnitude more differential PA genes. We will further discuss MAAPER below and focus on the comparison between Infernape and other methods first. A large proportion (96%) of the differential PA genes identified by Infernape was shared by at least one of the other methods (Fig. 2K). Infernape identified significantly more shared differential PA genes than Sierra, scAPA, and scAPAtrap (Fig. 2L; Supplemental Fig. S2E). These findings suggest that Infernape is well balanced between sensitivity and accuracy for identifying differential PAs.

MAAPER reported 5705 differential PA genes in total, and 80% (4592) of them were not identified as significant by Infernape or any of the other four tested methods (Supplemental Fig. S2E,F). Conversely, 544 out of 581 (94%) differential PA genes identified by Infernape were also identified by MAAPER (Supplemental Fig. S2F). When manually inspecting coverage plots and splice-junction reads, the Infernape-identified but MAAPER-missed differential PA events were found to be true differential PA genes (Supplemental Fig. S2G,H). These results indicate that Infernape identified bona fide differential PA events.

We next investigated MAAPER-specific signals (5161) to determine whether they were true PA changes. Thirty-three percent (1712) of MAAPER-specific PA genes were not identified as multipeak genes by Infernape and hence were not tested for differential PA. Most strong signals in this category had far upstream PAs/peaks near the transcription start sites instead of the annotated 3′ UTRs. For example, the PA signals of *Tln1* in MAAPER came from abundance changes of the 5′ intronic peaks (Supplemental Fig. S2I). Forty-two percent (2154) of MAAPER-specific differential PA genes were further filtered out by Infernape owing to low expression (detected in <5% of cells). For example, the coverage plot for *Acadl* shows two 3′-UTR peaks identified by both methods, but both peaks express in very few neurons (<1%) (Supplemental Fig. S2J). The remaining 25% of MAAPER-specific differential PA genes had large adjusted *P*-values (>0.05) in Infernape. The top MAAPER hits in this category showed high *P*-values and low effect size (measured with |MPRO|) in Infernape (Supplemental Fig. S2K). For example, the MAAPER-specific signal in *Hnrnpu* was caused by peak/PA variation in the first exon/intron, whereas the peaks/PAs in the annotated 3′ UTRs remain unchanged (<3%) (Supplemental Fig. S2L).

The far-upstream intronic peaks (PAs) identified by MAAPER were intriguing, and we further analyzed those regions using the E14.5 bulk RNA-seq data set. We counted reads in a 40-bp window upstream of each PA and found that 55.1% of the MAAPER-identified intronic PAs did not show any read counts compared with 10.3% for the MAAPER-identified PAs that were in annotated 3′ UTRs (Supplemental Fig. S2M), suggesting that a significant fraction of these intronic PAs identified by MAAPER were minor or not expressed. When we restricted peaks to annotated 3′ UTRs and their upstream 200-bp regions, MAAPER identified 30% fewer differential PA events, whereas the number of MAAPER–Infernape shared signals did not change as much (−4%). These results suggest that Infernape identifies biologically robust PA changes, whereas MAAPER is sensitive in PA detection. In summary, Infernape strikes a balance of sensitivity and accuracy in calling cell type–specific PAs from scRNA-seq data.

### scRNA-seq and single-nucleus RNA-seq uncover divergent PA patterns

**Figure 3.**
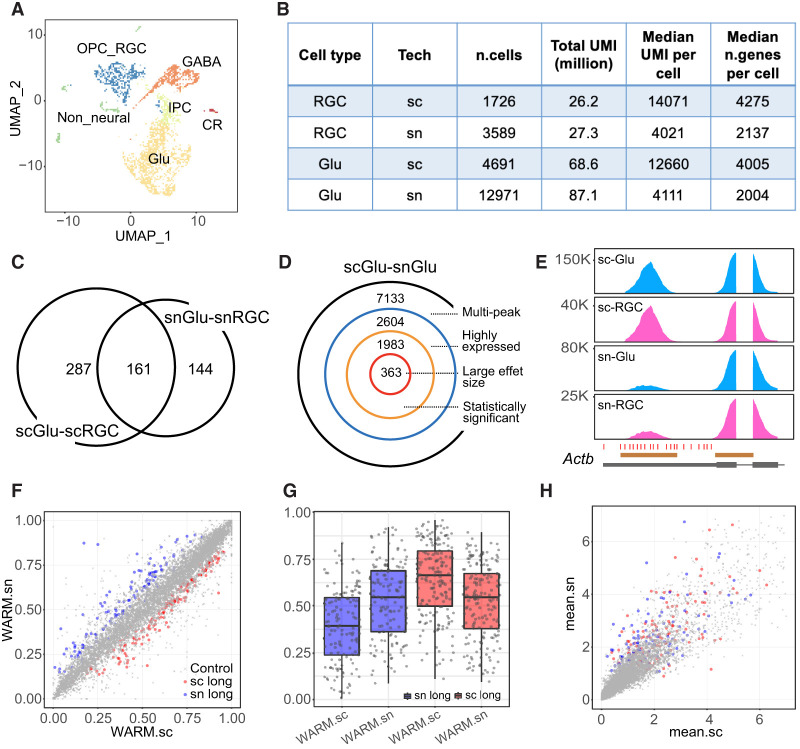
Differential PA discovery between scRNA-seq and snRNA-seq data. (*A*) UMAP showing six main cell types in the E18.5 mouse brain scRNA-seq data. (Glu) Glutamatergic neurons, (GABA) GABAergic interneurons, (IPC) intermediate progenitor cells, (OPC_RGC) oligodendrocyte precursor and radial glial cells, (CR) Cajal–Retzius cells. (*B*) Summary statistics for E18.5 mouse brain scRNA-seq and snRNA-seq data showing comparable total UMIs between two different data sets for both RGC (OPC_RGC) and Glu cells. (*C*) Venn diagram showing the numbers of significant differential PA genes between scGlu-scRGC (448 genes) and snGlu-snRGC (305 genes). (*D*) Decomposition of the number of significant differential PA genes for the Glu population between scRNA-seq and snRNA-seq (scGlu–snGlu). (*E*) Coverage plot of *Actb*, one of the top significant differential APA genes in the comparison of scRNA-seq versus snRNA-seq. Annotated PAs (red ticks) and peak regions (brown bars) by Inferenape are also shown. (*F*) Scatter plot showing WARM values for each transcript in the comparison of scGlu versus snGlu. Nonsignificant transcripts are labeled in gray, and significant transcripts are colored red (lengthening in scRNA-seq) or blue (lengthening in snRNA-seq). (*G*) Box plots showing the distribution of WARM values for significant transcripts in single-cell and single-nucleus data sets, respectively. The color codes are consistent with that in *F*. (*H*) Scatter plot showing average scaled gene expression for each transcript in *F*. The shape and color codes are the same as *F*.

Single-nucleus RNA-seq (snRNA-seq) has been increasingly used for highly multiplexed assays and postmortem tissues. We compared scRNA-seq and snRNA-seq data sets in identifying differential PA usage. Both single-cell and single-nucleus data sets (E18.5 mouse dorsal forebrain) were collected by 10x Genomics on the Chromium platform, and the main cell types showed comparable read depths (Fig. 3A,B; Supplemental S3A,B). Specifically, we identified main cell clusters in scRNA-seq data, annotated cell types with known marker genes (Fig. 3A; Supplemental Fig. S3C), and transferred cell-type labels from the scRNA-seq data set to the snRNA-seq data set (Supplemental Fig. S3C,D). Although the total number of high-quality cells is about twice in the snRNA-seq data than in the scRNA-seq data, the proportions of cell types are comparable (Supplemental Fig. S3A). scRNA-seq data show a higher number of expressed genes and normalized UMI counts per cell (Fig. 3B; Supplemental Fig. S3B), but overall, the total UMI counts are comparable between scRNA and snRNA data sets for major cell types (Fig. 3B).

We focused on differential PA events between glutamatergic neurons (Glu) and RGCs. We identified 448 differential PA events in the scRNA data set and 305 events in the snRNA data set, among which 161 events were shared (Fig. 3C; Supplemental Tables S3, S4). We next compared the Glu single-cell (scRNA-seq; scGlu) and Glu single-nucleus (snRNA-seq; snGlu) populations under a series of filtering criteria and found 363 differential PA events (scGlu–snGlu) (Fig. 3D; Supplemental Table S5). Similarly, there were 385 differential PA events between the RGC single-cell (scRGC) and RGC single-nucleus (snRGC) data sets (scRGC–snRGC) (Supplemental Fig. S3E–G; Supplemental Table S5), with 176 events overlapping those of the scGlu–snGlu comparison. Coverage plots for *Actb* confirmed the difference between the scRNA and snRNA data sets in both Glu and RGC cells (Fig. 3E). These results suggest that the scRNA and snRNA data showed a method-specific PA difference irrelevant to Glu or RGC cell types.

To determine whether the sc/snRNA-seq differential PA events were confounded by differential gene expression between the single-cell and single-nucleus data sets, we investigated the association between PA usage (3′-UTR lengthening/shortening) and gene expression levels (Fig. 3F–H). Genes with differential sc/snRNA-seq APA or IPA did not show biased expressions between the two data sets (Fig. 3F,H; Supplemental Fig. S3H,I). Lengthening APA signals in the scRNA-seq data tend to have even longer 3′ UTRs (larger WARM values) than those in the snRNA-seq data (Fig. 3F,G). We further compared reference transcript lengths, 3′-UTR lengths, and the GC contents of 3′ UTRs, and none of the three parameters showed a significant difference (Supplemental Fig. S3J), suggesting that the differential PAs reflect either mRNA export from the nucleus or a bias of sc/snRNA-seq protocols. These observations suggest that scRNA-seq and snRNA-seq uncover overlapping and divergent PA patterns that are not confounded by differential gene expression.

### Dorsal excitatory and ventral inhibitory neurogenesis trajectories share overlapping PA changes

**Figure 4.**
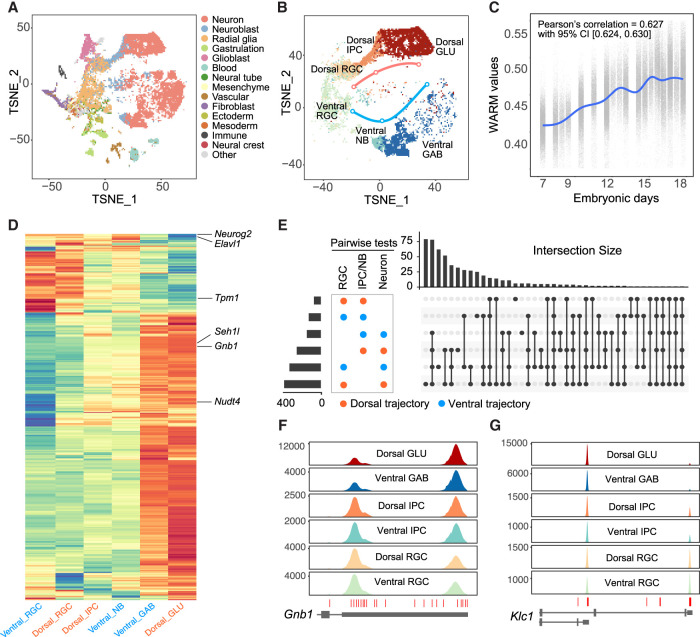
Dynamic PAs during dorsal excitatory and ventral inhibitory neurogenesis. (*A*) t-SNE plot showing the main cell types of the developing mouse brain from E7.5 through E18.5. Data were replotted from a previous study using original coordinates and cell-type labels (La Manno et al. 2021). (*B*) Neurogenesis trajectories for dorsal excitatory and ventral inhibitory neurons, respectively. (GLU) Glutamatergic neurons, (GAB) GABAergic neurons, (NB) neuroblast. (*C*) WARM values of single cells across sampling time showing significant 3′-UTR lengthening in brain development. (CI) Confidence interval. (*D*) Heat map of WARM values (within-UTR-level tests) showing shared APA events among comparisons for cell types in dorsal excitatory and ventral inhibitory neurogenesis trajectories. (*E*) UpSet plot for differential APA events showing shared changes among comparisons for cell types in dorsal excitatory and ventral inhibitory neurogenesis trajectories. (*F*) Coverage plot for *Gnb1* showing that Glu and GABA neurons tend to use the distal PA. (*G*) Coverage plot for *Klc1* IPA showing that Glu and GABA neurons primarily use the proximal PA.

Cortical inhibitory and excitatory neurons are generated in the ventral and dorsal germinal zones, respectively, and populate the neocortex through distinct molecular and migratory paths. We asked whether PA changes in the inhibitory and excitatory neuron lineages show overlapping or distinct patterns. To address this question, we reanalyzed 73,346 single cells from the developing mouse brain that spans E7.5 to E18.5 (Fig. 4A; La Manno et al. 2021). We identified the dorsal excitatory (RGC–IPC–GLU) and ventral inhibitory (RGC–NB–GABA) neuronal lineages based on originally reported cell cluster-specific genes and sampling information (Fig. 4B; Supplemental Fig. S4A–G). The WARM values increase across time and suggest that the average 3′-UTR length significantly increases from E7.5 through E18.5 (Fig. 4C). We plotted WARM values of individual cells and found the 3′ UTR lengthened during neurogenesis in both lineages (Fig. 4D; Supplemental Fig. S4H; Supplemental Tables S6–S9). Gene Ontology analysis identified the enrichment of biological functions such as protein ubiquitination and transport for 3′-UTR lengthening genes, and the enrichment of protein transport genes was cross-validated using an independent data set (Supplemental Table S10; Di Bella et al. 2021). These results suggest that both excitatory and inhibitory neurons tend to use distal PAs compared with neural progenitors.

Unsupervised clustering showed two major groups of APA in both excitatory and inhibitory lineages representing 3′-UTR lengthening and shortening events (Fig. 4D; Supplemental Table S9). The significant APA events during embryonic dorsal excitatory and ventral inhibitory neurogenesis largely overlapped, whereas more genes in both Glu and GABA neurons showed longer 3′ UTRs than in progenitor cells (Fig. 4D,E; Supplemental Tables S7, S9), such as *Gnb1* (Fig. 4F). In contrast, analyses of IPA events showed that progenitors and neurons in both lineages did not show a preference for proximal or distal PAs (Supplemental Fig. S4I; Supplemental Tables S8, S9). For example, the proximal PA of *Klc1* was predominantly used by both excitatory Glu and inhibitory GABA neurons (Fig. 4G). These results indicate that PA changes are substantially shared by excitatory and inhibitory neurogenesis processes, suggesting that cell proliferation has a predominant influence on PA usage.

Our previous study showed that the apical progenitors display temporal gene signatures during cortical neurogenesis (Ruan et al. 2021). To determine whether RGCs show temporal PA usage, we subset the RGCs based on sampling date. The results suggest that the WARM values increased in RGCs during development (Supplemental Fig. S4J) and cells in the G_2_/M phases show significantly higher WARM values than cells in the G_1_ or S phases (Supplemental Fig. S4K). These results suggest that RGCs express longer 3′ UTRs over time.

### Association of RBP expression with differential APA

**Figure 5.**
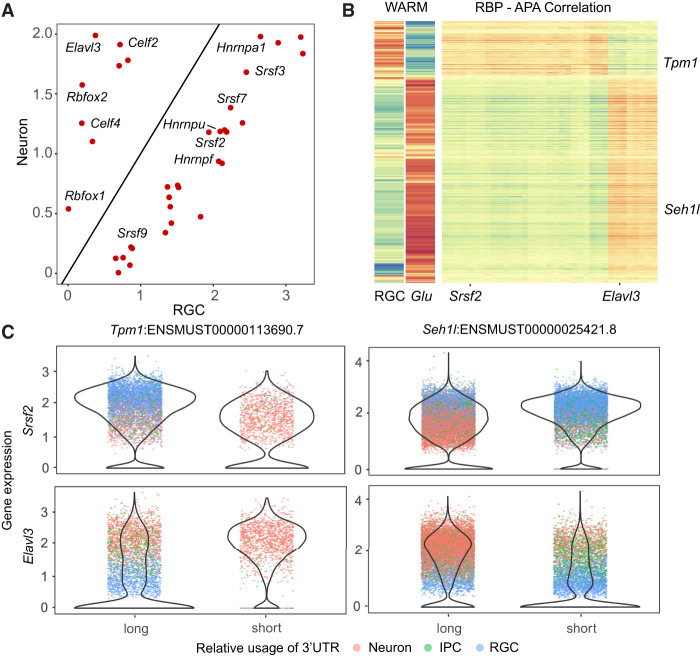
Differential APA and RBP expression in cortical neurogenesis. (*A*) Scatter plot showing differential expression of selected RBPs between RGCs and neurons. RBPs were selected only if their expression levels were significantly correlated or anticorrelated with 3′-UTR length changes. (*B*, *right* panel) Heat map showing the correlation of RBP expression levels and 3′-UTR lengths. APA signals were defined as transcripts with significant 3′-UTR length changes between RGC, IPC, and neurons. RBP genes were selected if (1) they were expressed in at least 10% of cells and (2) at least 10% of APA signals had a Pearson correlation coefficient >0.1 and adjusted *P*-values <0.05. Transcript names and orders are the same as in Figure 4D, and dorsal Glu and RGC cells were replotted to indicate the 3′-UTR length changes. (*C*) Violin plots showing the distribution of RBP transcript levels per cell for both high-WARM and low-WARM cell groups. Each dot represents a cell and is colored by cell types. Here we show four pairs of RBP and APA association between RBPs (rows for *Srsf2* and *Elavl3*) and transcripts with significantly differential APA (columns for *Tpm1* and *Seh1l*).

APA is regulated by RNA sequences surrounding the PAs sites and their interactions with RBPs. To understand the regulatory mechanisms of APA in cortical neurogenesis, we compared the differential RBP expression and their correlation with the 3′-UTR lengths across individual cells in the developing mouse brain data set (Fig. 4A). The RBPs that showed a significant correlation with APA usage separated into two main groups: *Celf2*, *Celf4*, *Elavl3*, and *Rbfox1/2* showed higher expression in neurons and correlated with longer 3′ UTRs, whereas *Hnrnpa1*, *Hnrnpf*, *Srsf2*, *Srsf3*, *Srsf7*, and other RBPs showed higher expression in neural progenitors and negatively correlated with 3′-UTR lengths (Fig. 5A,B; Supplemental Fig. S5A; Supplemental Table S11). We ectopically expressed *ELAVL3* in HEK293FT cells and validated APA lengthening in 57 genes such as *Gnb1*. Although the RBP–APA correlation was confounded by cell types (Fig. 5C), these results suggest that a subset of the RBPs might regulate PA usage in cortical neurogenesis.

### Cell class–specific PAs in the adult mouse brain

**Figure 6.**
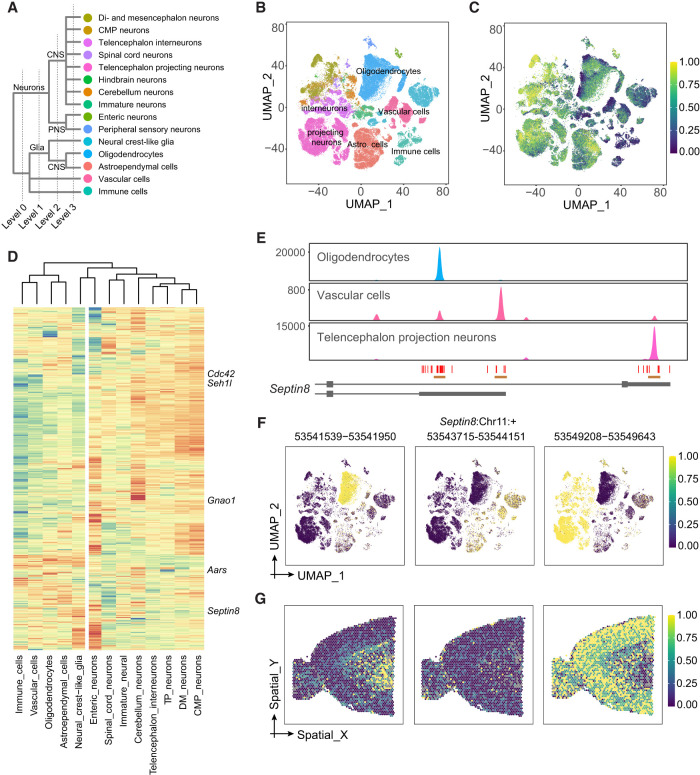
Cell class–specific PAs in the adult mouse brain. (*A*) Major cell classes in the adult mouse brain data set (Zeisel et al. 2018), showing four levels of hierarchy. (*B*) UMAP showing main level-3 cell types replotted from a previous study (Zeisel et al. 2018). The clusters are color-coded the same way as cell types in *A*. (*C*) UMAP showing transcriptome-wide average WARM values across cells. For each cell, the WARM values over all multipeak 3′ UTRs were averaged using equal weights. (*D*) Heat map showing cell type–level WARM values for significant differential APA transcripts among level-3 cell types. The dendrogram shows unsupervised clustering for cell types. Selected genes are labeled. The vertical white reference line indicates the separation of neurons and nonneuron cells. (*E*) Coverage plots of *Septin8* for oligodendrocytes, vascular cells, and telencephalon projecting neurons. The three peaks and associated PAs are indicated. The cell-type color codes are the same as *A*,*B*. (*F*) Relative expression patterns of three *Septin8* PAs identified by Infernape. The colors indicate the UMI counts proportion within each cell for the indicated peaks. (*G*) Expression of three *Septin8* PAs in the brain. The expression levels were standardized to [0, 1].

To further classify PA usage among brain cell types, we reanalyzed 146,676 cells from the adult mouse central nervous system (Fig. 6A,B; Zeisel et al. 2018). Through hierarchical comparison (Methods), we identified 2184 differential PA events out of 8519 multipeak 3′ UTRs (Supplemental Tables S12, S13). We overlaid the average WARM value per cell on UMAP to determine 3′-UTR length changes. We found that when using gene-expression-weighted average WARM, the oligodendrocytes showed longer 3′ UTRs than the unweighted average WARM (Fig. 6C; Supplemental Fig. S6A). Further investigation showed that the *Plp1* transcript, which was enriched in oligodendrocytes (Supplemental Fig. S6B,C), has a much higher expression level than other transcripts and dominated the weighted average WARM. As a comparison, weights across telencephalon projecting neurons are not dominated by a single or small set of genes (Supplemental Fig. S6C).

We performed pairwise comparisons in a hierarchical way based on the cell-type taxonomy (Fig. 6A,B; Supplemental Tables S12–S14). We identified differential PA events between neurons and nonneurons (level 0). Then within each of the level-0 cell types, we performed pairwise comparisons among all level-1 cell types. We repeated this procedure up to level 3, combined all differential PA events, and performed unsupervised clustering based on WARM values. One example of a cell type–defining PA event is in *Septin8*, for which three distinct peaks identified by Infernape were enriched in oligodendrocytes, nonoligo-nonneurons, and neurons, respectively (see below) (Fig. 6E,F; Supplemental Fig. S6D).

We examined the association between 3′-UTR lengths and gene expression levels. To eliminate cell-type confounding effects, we focused on the oligodendrocytes, a relatively transcriptome-homogeneous cell group (Methods) (Supplemental Fig. S6E). Out of 1727 highly expressed multipeak genes, WARM values of 194 and 179 genes significantly show a positive and negative correlation with their normalized expression levels, respectively (Supplemental Fig. S6F–I), suggesting that the overall PA usage in oligodendrocytes is not indicative of mRNA capture/expression levels.

### Visualizing cell type–specific PAs in the adult brain

We applied Infernape to spatial transcriptomic data of the adult mouse brain (10x Genomics Visium) and sought to correlate cell type–specific PA events to brain structures. First, we transferred cell-type labels from scRNA-seq analyses (Fig. 6A,B) to Visium spots: Cell clusters of telencephalon interneurons were more granular than those of telencephalon projection neurons (Supplemental Fig. S7A,B). The transferred cell-type labels for Visium spots were validated with layer-specific markers (Supplemental Fig. S7C–T). We identified and quantified peaks corresponding to PAs using the Visium data and constructed a peak-by-spot count matrix, based on which the differential PA events were measured and tested. The average WARM values per spot showed distinct patterns between the dorsal cortex and ventral brain regions (Supplemental Fig. S6J). The three PAs of *Septin8*, representing cell class–specific signals, displayed distinct patterns (Fig. 6G). These results suggest that we can identify cell type–specific and spatially resolved PA events in the brain.

**Figure 7.**
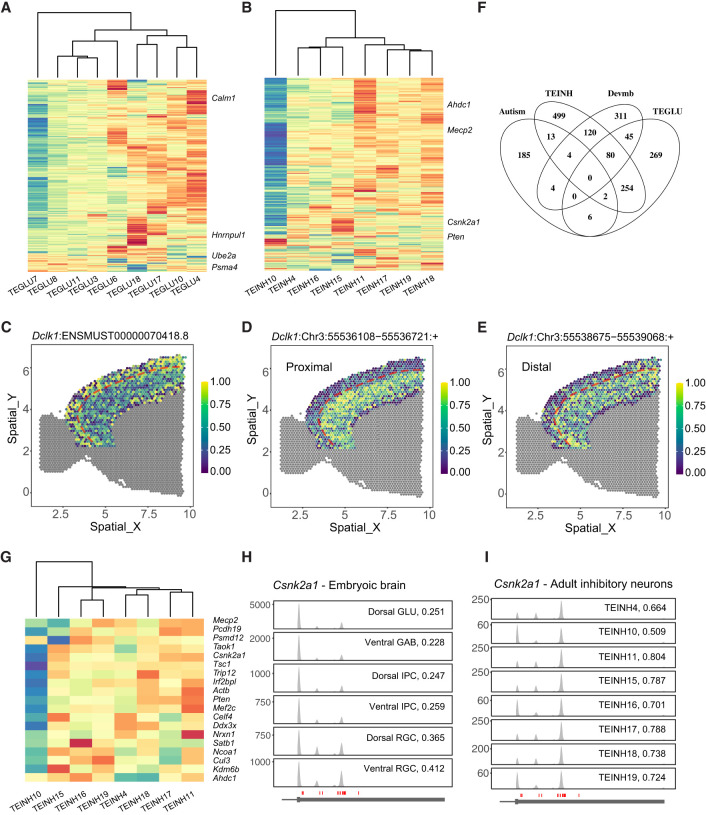
Cell type–specific PAs in the mouse brain. (*A*) Heat map showing cluster-level WARM values for significant differential PA transcripts among main telencephalon glutamatergic neuron clusters (TEGLU) (see also Supplemental Tables S15, S18). (*B*) Heat map showing cluster-level WARM values for significant differential PA transcripts among main telencephalon inhibitory neuron clusters (TEINH) (see also Supplemental Tables S16, S18). (*C*–*E*) APA patterns of transcript *Dclk1*:ENSMUST00000070418.8 in cortical layers. (*C*) WARM values per spot. (*D*,*E*) Relative expression of two peaks on this transcript, respectively. The dashed reference line indicates the boundary between layers II/III and IV. (*F*) Venn diagram for the following four gene lists: (1) SFARI autism-associated genes, (2) differential PA genes among TEINH, (3) differential PA genes among TEGLU, and (4) differential PA genes among developing mouse brain trajectories in the developing mouse brain data in Figure 4D. (*G*) Heat map showing cluster-level WARM values for the overlaps between autism-associated genes and significant differential PA genes among TEINH. (*H*) Coverage plot for *Csnk2a1* across cell types in the developing mouse brain. (*I*) Coverage plot for *Csnk2a1* across main adult TEINH.

We investigated PA characteristics within telencephalon projecting excitatory and inhibitory neuron subtypes and identified 657 and 975 significant differential PA events, respectively (Fig. 7A,B; Supplemental Fig. S7A,B; Supplemental Tables S15–S18). Measured by the WARM value per cluster, the PA patterns showed heterogeneity across telencephalon projection neuron types, especially for TEGLU7 (cortical layer II/III pyramidal neurons) and TEINH10 (Fig. 7A,B). The PA usage was informative for delineating cortical layers. For example, PA patterns of transcript *Dclk1*:ENSMUST00000070418.8 measured by per-spot WARM value highlight layer II/III in the neocortex (Fig. 7C–E; Supplemental Fig. S7S,T).

We further examined whether these differential PA genes were associated with neurological disorders such as autism (SFARI genes) and structural brain malformations (OMIM). We found 29 PA events in 25 genes associated with autism, among which eight PA events were differentially regulated during cortical neurogenesis and 17 PA events were dynamic between telencephalon inhibitory neuron types (Fig. 7F,G). For example, *Csnk2a1* displayed variable PA usage during neurodevelopment and across inhibitory neuron types in the adult brain (Fig. 7H,I); heterozygous mutations in *CSNK2A1* are associated with autism and Okur–Chung neurodevelopmental syndrome (Iossifov et al. 2014). In summary, these results nominate neuron subtype-specific PAs that may regulate gene expression in the mouse brain.

## Discussion

The 3′ UTR is a hotbed of regulatory sequences for mRNA dynamics (Tian and Manley 2017), and global 3′-UTR shortening has been associated with cell proliferation, tumorigenesis, and neurodevelopmental disorders (Sandberg et al. 2008; Ji et al. 2009; Mayr and Bartel 2009; Gennarino et al. 2015). The central nervous system expresses extended 3′ UTRs, and single-cell analysis uncovered cell type–specific PAs in certain brain cells such as GABAergic neurons and early mouse embryonic brain cells (Agarwal et al. 2021; Yang et al. 2021). It remains unclear how PAs are used and spatially distributed between cell types in embryonic and adult mouse brains. This study presents the Infernape analytical pipeline and shows its application in uncovering cell type–specific PAs in the mouse brain using scRNA-seq and spatial transcriptomics data.

Infernape uses a peak-centric approach, combines PA annotation with de novo PAS discovery, and introduces stringent statistical measures. To develop Infernape, we integrated and improved strategies from existing single-cell PA methods such as scAPA (Shulman and Elkon 2019), Sierra (Patrick et al. 2020), and MAAPER (Li et al. 2021c). Infernape improves PA identification and quantification by enhancing its accuracy in assigning read counts to cleavage sites. Specifically, overlapping peaks were decomposed using a Gaussian mixture model, which expands upon the capabilities of scAPA by accommodating more than two overlapping peaks. Inspired by MAPPER, we used a peak filtering technique that determines the distance between a peak mode and its corresponding PA based on single-PA–single-peak genes. This data-driven approach effectively links peaks with annotated PAs and facilitates the search for de novo PAS. Furthermore, to address the false discoveries arising from overdispersion in chi-square tests, we introduced the Dirichlet-multinomial test in our differential PA calling. Additionally, we introduced WARM and MPRO, which allowed an unrestricted number of PAs to summarize and rank effect sizes, overcoming the limitation of the static binary proximal-distal PA models. These procedures together enhanced the sensitivity and accuracy of Infernape in calling differential PAs.

Infernape uncovered bona fide PA events in the developing brain and minimized the impact of internal priming artifacts. Association analysis of RBP expression and 3′-UTR lengths at the single-cell level uncovered RBPs that correlate with PA usage, whereas the confounding effect of cell type–specific gene expression remains to be addressed. We further uncovered cell class–specific and cell type–specific PA events in the adult mouse brain and projected such PA signals onto brain structures. These results suggest that PA differentiates brain cell types and tunes gene expression.

We were motivated to compare scRNA-seq and snRNA-seq data in PA discovery because snRNA-seq has been increasingly used for high-throughput studies, especially for postmortem human tissues (Rozenblatt-Rosen et al. 2017; Cao et al. 2019). We uncovered divergent and method-specific PA events from scRNA-seq and snRNA-seq data sets generated by 10x Genomics, suggesting the difference was caused by either technical bias or differential subcellular distribution (or nuclear export) of transcripts.

The dorsal excitatory and ventral inhibitory neuronal lineages display divergent neurogenesis, migration, and differentiation features (Dehay and Kennedy 2007) but showed 3′-UTR lengthening and overlapping PA changes in both neurogenesis lineages based on the original cell-type annotations and sampling information from a previous study (La Manno et al. 2021). These results are consistent with previous reports that cell proliferation affects 3′-UTR lengths (Sandberg et al. 2008) and that neuronal genes express longer 3′ UTRs (Ji et al. 2009; Agarwal et al. 2021).

Our PA analyses of the adult mouse brain uncovered cell class–specific and cell type–specific PAs. First, neurons and nonneuron cells were well separated depending on the PA information (Fig. 6D). Second, we uncovered cell class–defining PA events such as the *Septin8* gene, which expressed three distinct PAs in oligodendrocytes, nonoligo-nonneuron cells, and projection neurons. The spatial distributions of *Septin8* PAs were indeed associated with the corresponding cell classes. Third, we uncovered PA events that delineated cortical layers (Fig. 7). These results suggest that differential PA usage plays a role in cell type–specific gene regulation.

Mutations in more than 100 genes have been reported to cause autism, and the convergent biological pathways have been actively studied. Our PA analyses suggest that at least 25 autism genes are regulated by cell type–specific PAs, among which *Mecp2*, *Csnk2a1*, and 17 other genes showed variable PAs between inhibitory neuron subtypes. This study presents Infernape to uncover PA usage from single-cell and spatial transcriptomic data, and nominates PAs for their potential functions in brain development.

## Methods

### Mouse protocols and molecular experiments

Mouse protocols were reviewed and approved by the University of Chicago Institutional animal care and use committee. The dorsal forebrains of E14.5 *Tg(Tbr2:EGFP)* and *Tg(Tubb3:EGFP)* Bac transgenic mouse lines were used to isolate cells for bulk RNA-seq as described previously (Zhang et al. 2016; Yang et al. 2023). Briefly, the *Eomes:EGFP* Bac transgenic line labels IPCs and excitatory neurons at E14.5 as shown before, likely because of the slow degradation of EGFP (Zhang et al. 2016). Thus, the *Eomes:EGFP*-negative cells are mostly E14.5 RGCs. Raw reads were trimmed and aligned to mouse mm10 with STAR aligner (Dobin et al. 2013), and PA analysis of bulk RNA-seq was performed with REPAC (Imada et al. 2023). RNA in situ hybridization was performed as previously described (Schaeren-Wiemers and Gerfin-Moser 1993). Briefly, digoxigenin-labeled antisense RNA probes were transcribed in vitro from the specific segment of different *Gnao1* isoforms cloned in the pGEM-T vector. The primers for cloning *Gnao1* isoform-specific sequences were as follows: forward primer TAGCATGACCTTTGGCCTTT and reverse primer GGCTGGGTGAATTGCTTCTA for the Gnao1_Neuron probe, and forward primer GCAGAGGTGTGGAACAGCA and reverse primer GCATTCTCAGGGCTTGTCAT for Gnao1_NPC probe. Labeled slices were imaged using a Zeiss Axio Imager widefield microscope.

### Peak detection

The initial step in Infernape involves peak detection to identify potential polyadenylation sites. To mitigate PCR amplification bias, we use deduplicated UMI counts to create a table of raw observed read counts for each genomic position within every gene. Next, to determine the peak modes, we apply a Gaussian kernel smoother to the raw counts and generate a noise-reduced curve. The peak modes are then identified as the local maxima of this curve.

To enhance accuracy, Infernape incorporates a filtering-and-merging process to selectively retain peaks located within the 3′-UTR region, exclude minor peaks, and merge peaks that are in close proximity. Specifically, Infernape excludes peaks with either fewer than 10 reads or with ≤5% reads of the largest peak mode within the 3′-UTR region of the corresponding gene. If two peaks are within 50 bp of each other, they are merged into a single peak centered at the mean of the original two peak modes. Given our primary interests in APA and IPA, we focus on peaks with modes within the extended reference 3′ UTRs (Agarwal et al. 2021).

### Peak fitting

In step 2, once the raw peak modes have been detected, we proceed to refine the peak mode locations and their spread using a local parametric Gaussian density model of the read counts, building upon the approach used in scAPA (Shulman and Elkon 2019) and Sierra (Patrick et al. 2020). A significant challenge arises when certain PAs are in close proximity, resulting in heavily overlapped peak regions. To tackle this challenge, we first classify the raw peak modes as isolated or overlapped based on the presence of at least one other raw peak mode within a 300-bp radius. In the case of overlapping peaks, we designate a peak cluster comprising all peaks overlapped with at least one other peak within the same cluster.

For isolated peak regions, following the methodology of Sierra (Patrick et al. 2020), we fit the local region by using a Gaussian kernel with least square regression. For peak clusters with overlapping peak modes, we fit the region using a K-component Gaussian mixture model through the EM algorithm, where K denotes the number of peaks within a given peak cluster. To enhance the accuracy of the fitting process, we temporarily retain the non-3′-UTR peak if a peak cluster contains peaks located outside the 3′ UTRs. The non-3′-UTR peaks are not considered in the subsequent analysis.

### Peak annotation and filtering

In step 3, we leverage established PA annotations to refine our peak selection and filter out peaks that are unlikely to correspond to authentic polyadenylation sites. A key step is determining the distance between a peak mode and its corresponding PA. To achieve this, we draw inspiration from MAAPER (Li et al. 2021c), which uses single-PA–single-peak genes as controls to estimate the distance between peak modes and their corresponding PAs. We use the PA reference by incorporating annotated PAs from PolyA_DB (v3) (Wang et al. 2018), PolyASite (2.0) (Herrmann et al. 2020), and GENCODE (M25), as was performed in a previous study (Agarwal et al. 2021). We select genes that have only one known PA and only one peak mode detected in the earlier steps. To establish a reliable measure, we construct a standard interval for peak mode-PA distance (SID), which represents the 5% and 95% quantiles of the observed distances between the PA and peak modes in the single-PA–single-peak gene set. Leveraging the SID, we define three filtering rules: (1) the presence of at least one known PA within the SID; (2) the occurrence of at least one PAS, including canonical motifs A[A/T]TAAA and their variants such as TTTAAA, AAGAAA, AACAAA, TATAAA, AATGAA, AGTAAA, AATATA, CATAAA, ACTAAA, GATAAA (Li et al. 2021b), within a region shifted 20 bp upstream of the cleavage sies; and (3) the presence of a sequence of 13 consecutive adenosines, referred to as an A-stretch, within the SID. Peaks that satisfy either rule 1 or rule 2 but not rule 3 are retained for further analysis.

### Read counting

In step 4, we assign each observed UMI count to a filtered and annotated peak, creating a peak-by-cell count matrix as the observed data for subsequent analyses. For each peak, denoted as peak *j*, we define its peak region as μ_*j*_ ± 3σ_*j*_, where μ_*j*_ represents the estimated peak location, and σ_*j*_ represents the estimated spread obtained in step 2. When determining whether a read overlaps with a specific peak region of interest, we consider only matched positions, identified by the CIGAR operation = M. In cases in which multiple reads share the same UMI barcode, we retain only the median locations. If a read overlaps with multiple peak regions, we assign it to the peak that possesses the maximum posterior probability, calculated from our fitted K-component Gaussian mixture model in step 2.

### Statistical testing

To identify differential PA events across cell types, we use a gene-level test that accommodates multiple PAs within the same gene while simultaneously comparing across multiple cell types. To mitigate the risk of false positives, we use a combination of a chi-square test and a Dirichlet-multinomial test. This approach effectively addresses both biological and technical noise inherent in scRNA-seq data.

We consider the observed count ***X***_*ig*_ = (*X*_*ig*1_, · · · *X*_*igJ*_) for cell *i* and gene *g*, where *J* represents the number of PAs of gene *g*. We assume that ***X***_*ig*_ follows a multinomial distribution $X_{ig}∼\mathrm{Multinomial}(n_{ig},\;p_{ig})$, with *n*_*ig*_ denoting the total UMI count and ***p***_*ig*_ representing the proportion vector across *J* peaks. Existing differential PA analysis methods rely on chi-square tests, which assume homogeneity within and across cell types under the null hypothesis of no differential PA events across cell types. In other words, it assumes that ***p***_*ig*_ ≡ ***p***_*kg*_ ≡ ***p***_*g*_ for any cell *i* of cell-type *k* under the null. However, this assumption overlooks biological variability across cells and can yield an inflated number of false positives.

To account for within-cell-type heterogeneity, we introduce a Dirichlet distribution to model the randomness in ***p***_*ig*_ within each cell type: $p_{ig}∼\mathrm{Dirichlet}(\alpha_{kg})$ for any cell *i* of cell-type *k*. We perform a likelihood ratio test to evaluate the null hypothesis: *H*_0*g*_:**α**_1*g*_ = · · · = **α**_*Kg*_ for each gene (DM test). The rejection of *H*_0*g*_ suggests either a differential PA event or a change in the randomness level across cell types. Therefore, to ensure the identification of differential PA events, we consider the maximum of the *P*-values from both the chi-square test and the DM test as the final *P*-value for each gene.

### Weighted average relative mode position

WARM quantifies PA usage by measuring the average relative position of all PA-associated peaks. For a multipeak transcript/gene, denote the relative position of the most proximal/distal peak mode as zero or one, respectively, and other peaks are assigned numbers between [0, 1]. In the case that all peaks are from the same 3′ UTR, relative positions are linearly interpolated according to mode positions on the genome. In the case that peaks are from transcripts with different 3′ UTRs, relative positions are evenly interpolated within [0, 1] based on only the rank of the actual genome position. Specifically, assume that actual peak positions on the genome are *t*_*i*_, *i = 1, 2,* …, *n*. For peaks with *rank(t_i_) > 0* and *< n*, the relative position is calculated as *rank(t_i_)/n*. WARM is calculated as the average relative positions weighted by reads counts. The average WARM for all genes in a cell is a measure of global PA usage, and the delta WARM can summarize PA differences between two cell groups at single-gene or whole-transcriptome levels.

### Maximum difference in proportion change

To identify local PA changes in a given gene, MPRO quantifies PA by measuring the greatest contrast in proportion change among all peak pairs. Assuming the two cell groups in comparison to be the base and alternative (*alt*) group, proportion (*Prop*) is calculated over all peaks in a specific cell group. For one peak, proportion change is defined as *δ* = *Prop in base – Prop in alt*. For one peak-pair (*i, j*), where peak *i* is downstream from peak *j*, the difference in proportion change is defined as *dδ* = *δi* – *δj*. MPRO is calculated as *dδ* with the largest absolute value over all possible peak pairs. A positive/negative MPRO value implies that the base cell type tends to use more distal/proximal peaks than the alternative cell type, respectively. MPRO is sensitive in finding local differential PA events and is used as an effect size filter in Infernape.

### Determining final differential PA signals

Final differential PA signals between cell types are determined by combining statistical test results, expression levels, and effect sizes. The rules include (1) adjusted *P*-values by Benjamini–Hochberg correction of <0.05, (2) for each cell type in comparison, at least one peak expressed in >5% cells, and (3) |MPRO| > 0.2 (10% PA change for double peak/PA genes).

### APA–RBP expression association

The APA gene list was derived from the test results of dorsal RGC–IPC–GLU neurons versus ventral RGC–NB–GABA neuron lineages in developing mouse brain scRNA-seq data. We tested the hypothesis of Pearson correlation coefficient of 0 for each pair of the RBP gene (GO:0003723, https://www.informatics.jax.org) and the APA gene. We kept RBPs that had (1) a Pearson correlation coefficient >0.1 and (2) an adjusted *P*-value <0.05 in at least 15% of all APA genes.

### 3′-UTR relative length–gene expression association

We selected a subset of homogeneous cells, clusters MOL1-3, in the adult mouse brain data set (15,341 cells). In each MOL cell, we calculated WARM value and normalized UMI counts for each of 8372 multipeak 3′ UTRs. A 3′ UTR was discarded if the proportion of cells with NA WARM values (or not expressed) is >90%. After this filter, we have 1727 3′ UTRs left. We then calculated Kendal's tau correlation for each WARM-expression pair. The corresponding *P*-values (testing against the null of no correlation) were reported. A significant correlation was concluded if the adjusted *P*-value (Bonferroni correction) was less than 0.05. Three hundred seventy-three signals versus 1354 noise are shown in Supplemental Figure S6C.

### Hierarchical PA test

Cell types were identified hierarchically. As illustrated in Figure 6A and Supplemental Table S15, all cells were categorized into neurons or nonneurons (level 1); neurons were next categorized into CNS and PNS, whereas nonneurons were categorized into glia and nonglia (level 2). Cells were further grouped into major cell classes. Following this logic, a tree of taxonomy was formed so that ancestral and parental cell types can be traced. To avoid redundancy, we performed PA tests hierarchically. For a cell type *c*_*L*_, where the subscript *L* represents its taxonomy level, we first tested *c*_*L*_ versus a cell group including all non-*c*_*L*_ cells in *c*_*L* − *1*_. Next, we test *c*_*L-1*_ versus a cell group including all non-*c*_*L* − *1*_ cells in *c*_*L* − *2*_. This process was repeated until the root of the taxonomy tree was reached (*c*_*0*_ is assumed to be the cell type including all cells).

#### Benchmarking single-cell PA detection methods

We compare Infernape with single-cell PA calling methods including MAAPER (v1.1.1) (Li et al. 2021c), Sierra (v0.99.27) (Patrick et al. 2020), scAPA (v0.1.0) (Shulman and Elkon 2019), SCAPTURE (v1) (Li et al. 2021a), and scAPAtrap (v0.1.0) (Wu et al. 2021). We used the default parameters for peak or PA calling on mouse E14.5 scRNA-seq data and performed differential analysis between glutamatergic neurons and RGCs. To ensure consistency, all identified peaks from these methods were reannotated using the 3′-UTR annotation files used by Infernape.

#### ELAVL3 bulk RNA-seq

The coding sequence of human *ELAVL3* was amplified and inserted into pR008 under the pCAG promoter using Gibson assembly (NEB E2611L). The plasmid and pR008 (control) were transfected into HEK293FT cells (Thermo Fisher Scientific R70007), which were cultured at 37°C in DMEM (Gibco 10566024) supplemented with 10% fetal bovine serum (Gibco 26140079) in a humidified incubator with 5% carbon dioxide. For each well in a 12-well plate, 8 × 10^5^ cells were transfected in suspension with Lipofectamine 2000 (Thermo Fisher Scientific 11668019) and changed to fresh medium 4 h after transfection. Cells were dissociated 24 h after transfection, and EGFP-positive cells were isolated with flow cytometry and further processed for RNA extraction (Zymo Direct-zol, R2060) and RNA-seq (TruSeq stranded mRNA library prep kit, Illumina 20020594). Sequence processing and alignment were performed as described previously (Ruan et al. 2023), and differential PA events were analyzed using REPAC (Imada et al. 2023).

#### Published data used in the analysis

Developing mouse brain (La Manno et al. 2021) scRNA-seq data can be downloaded from the NCBI BioProject database (https://www.ncbi.nlm.nih.gov/bioproject/) under accession number PRJNA637987. Adult mouse brain (Zeisel et al. 2018) scRNA-seq data are available at the NCBI Sequence Read Archive (SRA; https://www.ncbi.nlm.nih.gov/sra) under accession number SRP135960. Spatial Visium and E18.5 mouse brain data sets are available at https://www.10xgenomics.com/resources/datasets. Bulk RNA-seq data for E14.5 *Tubb3:EGFP*-positive and *Eomes:EGFP*-negative cells (Yang et al. 2023) can be downloaded from the NCBI BioProject database (https://www.ncbi.nlm.nih.gov/bioproject/) under accession number PRJNA930469: SRR23308049, SRR23308050.

## Data access

The bulk RNA-seq data for *ELAVL3* ectopic expression and control in HEK293FT cells generated in this study have been submitted to the NCBI BioProject database (https://www.ncbi.nlm.nih.gov/bioproject/) under accession number PRJNA972714. Infernape software is available at GitHub (https://github.com/kangbw702/Infernape) and as Supplemental Code. The interactive differential PA Web portal can be accessed at https://zlab1.shinyapps.io/Infernape/.

## Supplementary Material

Supplement 1

Supplement 2

Supplement 3
