# Supplementary material for: Infernape uncovers cell type–specific and spatially resolved alternative polyadenylation in the brain
Source: Genome Res. 2023 Oct;33(10):1774–87. doi: 10.1101/gr.277864.123 (PMC10691540; doi:10.1101/gr.277864.123)
Supplement: Supplement 2 [file Supplemental_Figures.pdf]

Infernape uncovers cell-type-specific and spatially resolved alternative polyadenylation in the brain

Bowei Kang[1], Yalan Yang[1], Kaining Hu[1], Xiangbin Ruan[1], Yi-Lin Liu[2], Pinky Lee[1], Jasper Lee[1], Jingshu Wang[2,*], Xiaochang Zhang[1,3,*,†]

1. Department of Human Genetics, The University of Chicago, Chicago, IL 60637, USA
2. Department of Statistics, The University of Chicago, Chicago, IL 60637, USA
3. The Neuroscience Institute, The University of Chicago, Chicago, IL 60637, USA

[*]Correspondence: xczhang@uchicago.edu (X. Z.), jingshuw@uchicago.edu (J. W.)
[†]Lead contact

Supplemental Figure S1-S7 and Figure Legends

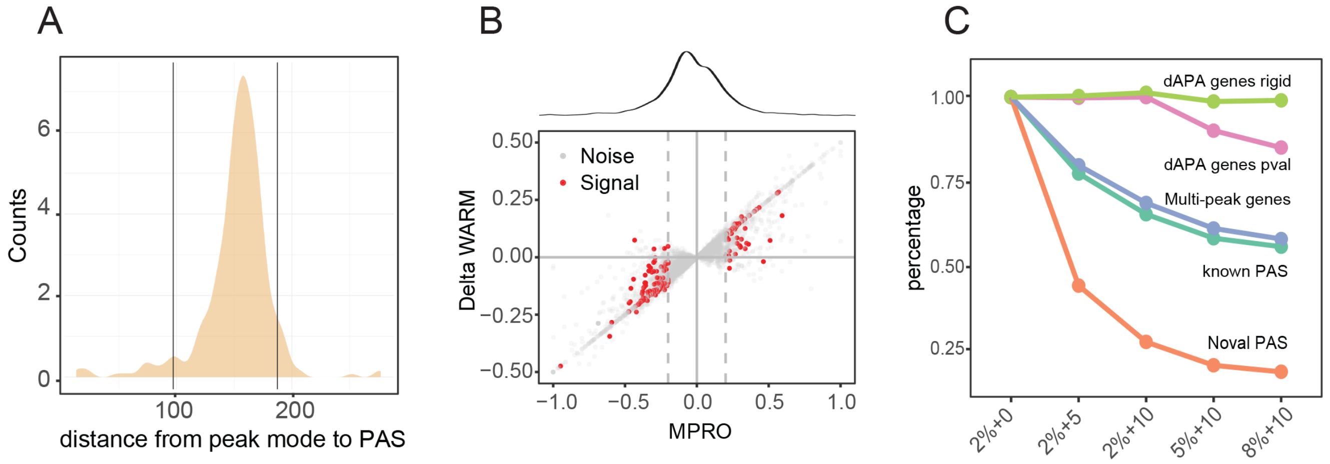

Figure S1. Features of Infernape. Related to Figure 1.

A. The distribution of distances from peak mode to the asscoaited PAs for single-PA-single-peak genes in the adult mouse brain data. The vertical reference lines represent 5% and 95% empirical percentiles, respectively.

B. Relationship between MPRO and delta WARM value, illustrated by the within-UTR level APA test result for radial glia cells versus neurons in the E14.5 mouse forebrain data. The density of MPRO is shown in the top panel. In the bottom panel, the solid reference lines represent the neutral APA case and the dashed reference lines represent +/- 20% cutoffs for MPRO.

C. Sensitibity analysis for Infernape. Scenarios are defined as 'relative cutoff + absolute cutoff' in peak calling. For example, "2%+5" means all raw peaks with read counts larger than max (2% of of max height in the associated gene, 5) will be kept for futher analysis. The results from the most flexible setting, "2%+0" is considered as baseline and is normarlized to be 100 percent. Other results are quantified proportionally. The measures quantified (from bottom to top) include: peaks associated with known PAs, peaks asscociated with *de noval* PAs, genes with more than one peaks, differential PA genes with adjusted p-value < 0.05, differential PA genes determined by the rigid rule of Infernape.

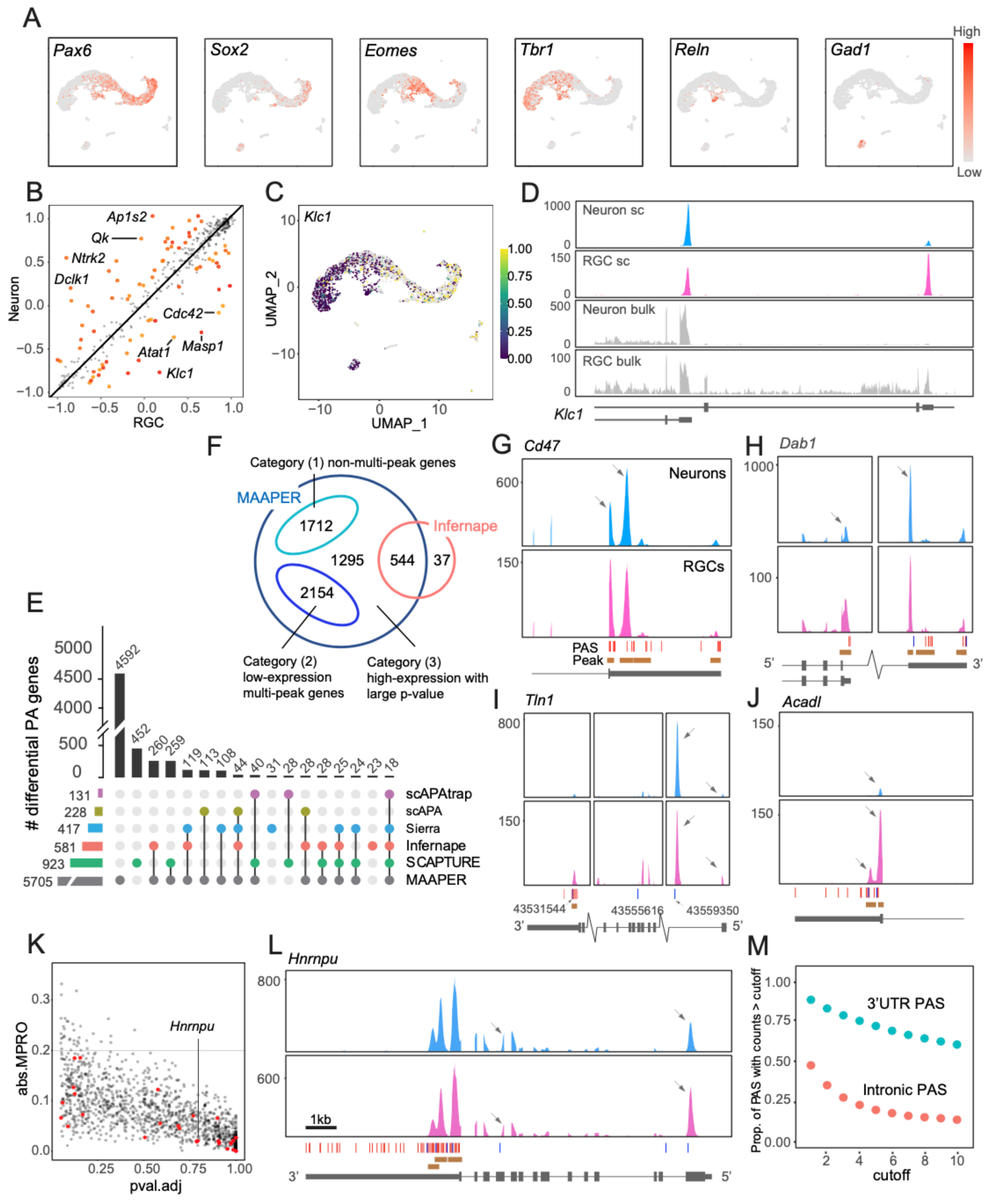

Figure S2. Comparison of Infernape and MAAPER and other PA packages. Related to Figure 2.

A. UMAP feature plots of E14.5 scRNA-seq data showing the expression of markers for RGCs (*Pax6*, *Sox2*), IPCs (*Eomes*), Layer VI and SP (*Tbr1*), Layer I (*Reln*), and inhibitory neurons (*Gad1*).

B. Scatter plot showing IPA test results. Each dot represents a gene and the x/y-axis represent WARM values for the two cell types in comparison.
C. UMAP showing that WARM values of the *Klc1* gene were higher in RGCs.
D. Coverage plot of the *Klc1* gene for Neurons and RGCs, showing both scRNA-seq (blue and pink) and bulk RNA-seq data (gray).
E. An UpSet plot showing differential PA genes identified by different methods when comparing RGCs and neurons in the E14.5 scRNA-seq data.
F. Venn diagram showing the number of shared and MAAPER- / Infernape-specific differential PA genes (both 3'UTR length change and IPA). To further investigate the difference between the two methods, MAAPER-specific differential PA genes were decomposed into three categories: (1) non-multi-peak genes in Infernape, (2) low-expression (peak detected in less than 5% of cells for either cell type) multi-peak genes in Infernape, and (3) high-expression (peak detected in more than 5% of cells for both cell types) multi-peak genes with significant p-values in Infernape. This categorization is used in following Fig.S2I-S2L.
G. Coverage plot of *Cd47* as an example of the Infernape-specific APA gene. From top to bottom, tracks 1 and 2 show de-duplicated read counts in E14.5 Neurons and RGCs, respectively. Track 3 shows PAs in integrated reference (red ticks). Track 4 shows the peak intervals estimated by Infernape. Track 5 shows the reference transcripts.
H. Coverage plot of *Dab1* as an example of the Infernape-specific IPA gene. Blue ticks in the PA track represent the PAs identified in MAAPER.
I. Coverage plot for an example of MAAPER-specific category (1) gene, *Tln1*. MAAPER identified only one PA in the 3'UTR region of *Tln1*, which is consistent with the single peak found in Infernape. The APA significance of *Tln1* in MAAPER comes from the abundance change between the PA in the first intron and the PA in the 3'UTR.
J. Coverage plot for an example of MAAPER-specific category (2) gene, *Acadl,* showing low gene expression in Neurons. Two 3'UTR PAs/peaks were identified by both methods, but both peaks were expressed in very few cells (<1%) in Neurons.
K. Scatter plot showing adjusted p-value versus effect size (absolute MRPO, Methods) for all category (3) signals identified by MAAPER. The top 25 hits with the smallest p-values are colored red.
L. Coverage plot for an example of MAAPER-specific category (3) gene, *Hnrnpu*. Coverage plot for *Hnrnpu* showing that the significant signal of this gene in MAAPER was mainly due to the peak identified in the first exon/intron, while the usage of peaks/PAs in the 3'UTR remained unchanged.
M. The proportion of MAAPER-identified PAs and corresponding read counts in a 40bp window upstream of each PA. The read counts were derived from bulk RNA-seq of the E14.5 RGCs and Neurons. Upstream intronic PAs (red) had substantially fewer reads than 3'end PAs (blue).

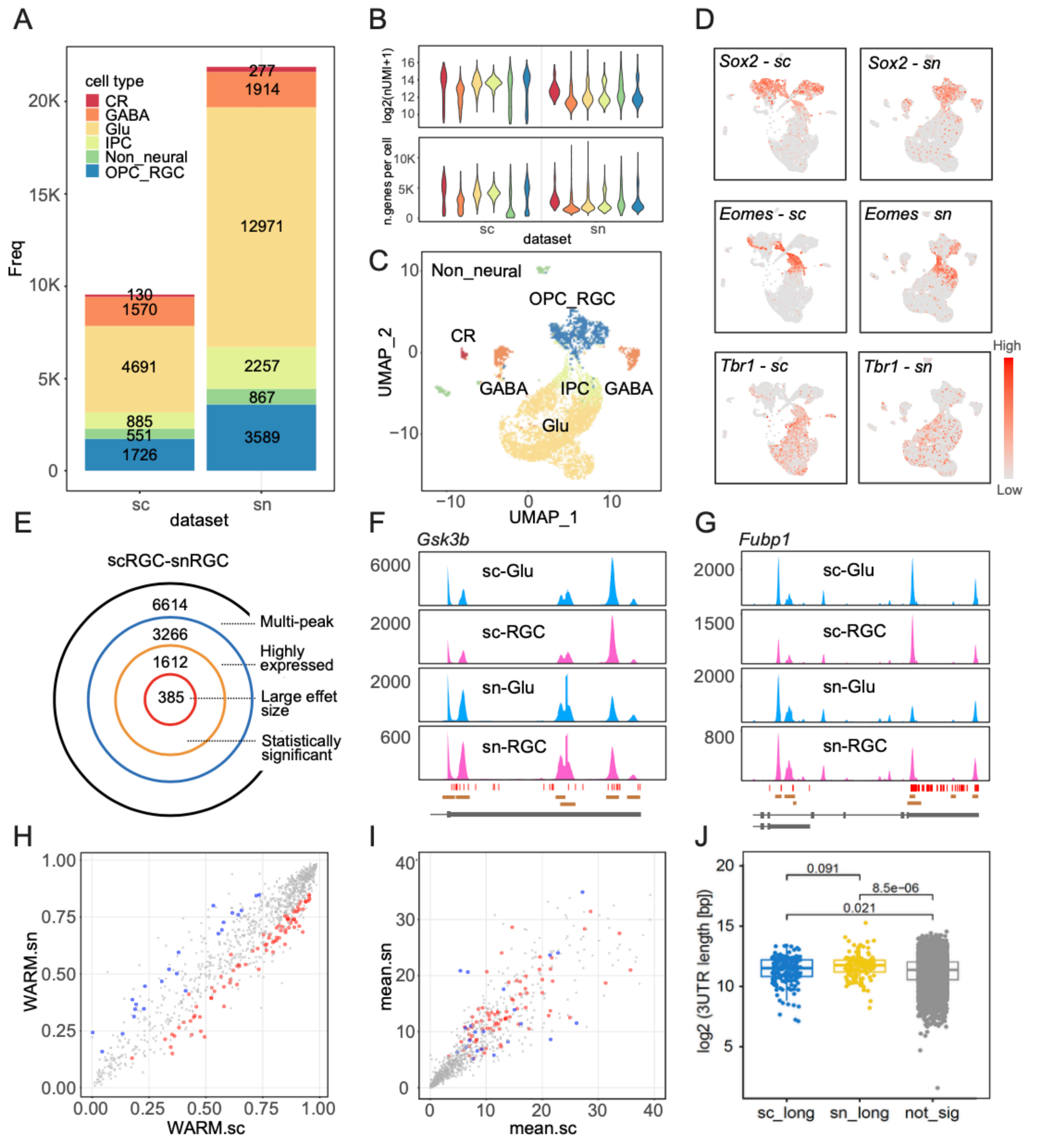

Figure S3. Differential PA discovery between scRNA-seq and snRNA-seq data. Related to Figure 3.

A. Stack bar plot showing the number of cells and proportions grouped by cell type for E18.5 mouse brain scRNA-seq and snRNA-seq data.
B. Violin plot showing the average number of read counts and expressed genes per cell for each cell type in E18.5 mouse brain scRNA-seq and snRNA-seq data.
C. UMAP showing six main cell types for E18.5 mouse brain snRNA-seq data. The cell type annotations were transferred from the scRNA-seq data in Fig.3A.
D. Feature plots of scRNA-seq (top) and snRNA-seq data showing marker genes for OPC_RGCs (*Sox2*), IPCs (*Eomes*), and *Tbr1*-positive Neurons.

E. Decomposition of the number of significant differential PA genes for the comparison of RGC populations between scRNA-seq and snRNA-seq data (scRGC-snRGC).
F. Coverage plots for *Gsk3b*, a top significant differential PA gene in the comparison performed in Fig.3D and S3E.
G. Coverage plots for *Fubp1*, a top significant differential PA gene in the comparison performed in Fig.3D and S3E.
H. Scatter plot showing WARM values for each multi-3'UTR gene in the comparison of RGC scRNA-seq versus RGC snRNA-seq. Non-significant genes are labeled in gray, and significant genes are colored in red (lengthening in scRNA-seq) or blue (lengthening in snRNA-seq).
I. Scatter plot showing average scaled gene expression for each gene in Fig.S3H. The color codes are the same with Fig.S3H.
J. Box plots showing that the lengths of reference 3'UTRs do not show a significant difference between Glu sc/sn-specific PA events (Wilcoxon test).

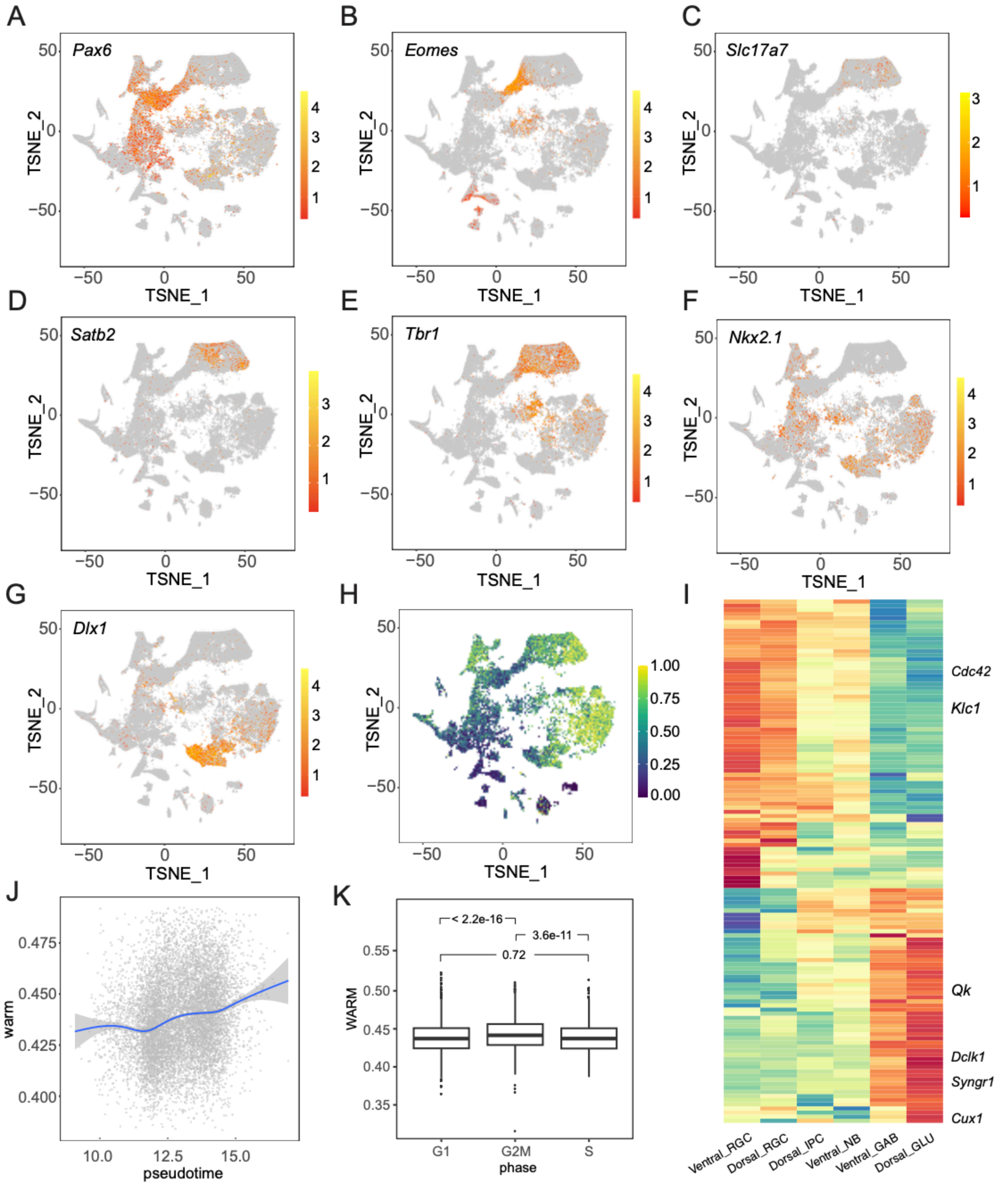

Figure S4. Dynamic PAs during neurogenesis. Related to Figure 4.

A-B. Feature plots showing marker gene expressions of the dorsal RGCs (*Pax6*) and IPCs (*Eomes*).

C-E. Feature plots showing *Slc17a7*, *Satb2*, and *Tbr1* expression marking the dorsal Glutermatergic neurons.

F-G. Feature plots showing *Nkx2.1* and *Dlx1* expression marking the ventral inhibitory neuron lineage.

H. t-SNE plot (Fig.4A) overlaying average WARM values.

I. Heatmap of WARM values for IPA events.
J. Scatter plot and trend line showing average WARM value across peudotime for Dorsal RGCs.
K. Boxplot showing average WARM value versus predicted mitotic phases of dorsal RGCs (t-test).

A

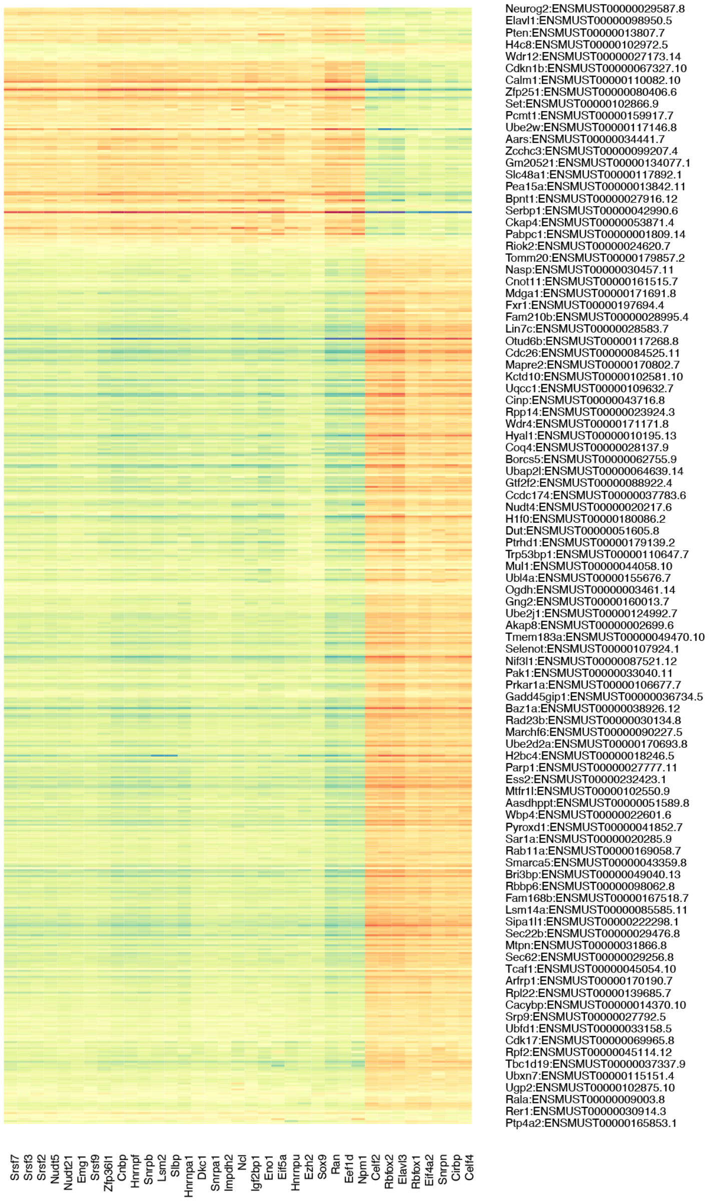

Figure 5. Correlation of APA and RBP expression in cortical neurogenesis. Related to Figure 5.

A. Heat map showing a subset of RBPs (bottom) that are significantly associated with differential PA usage during cortical neurogenesis.

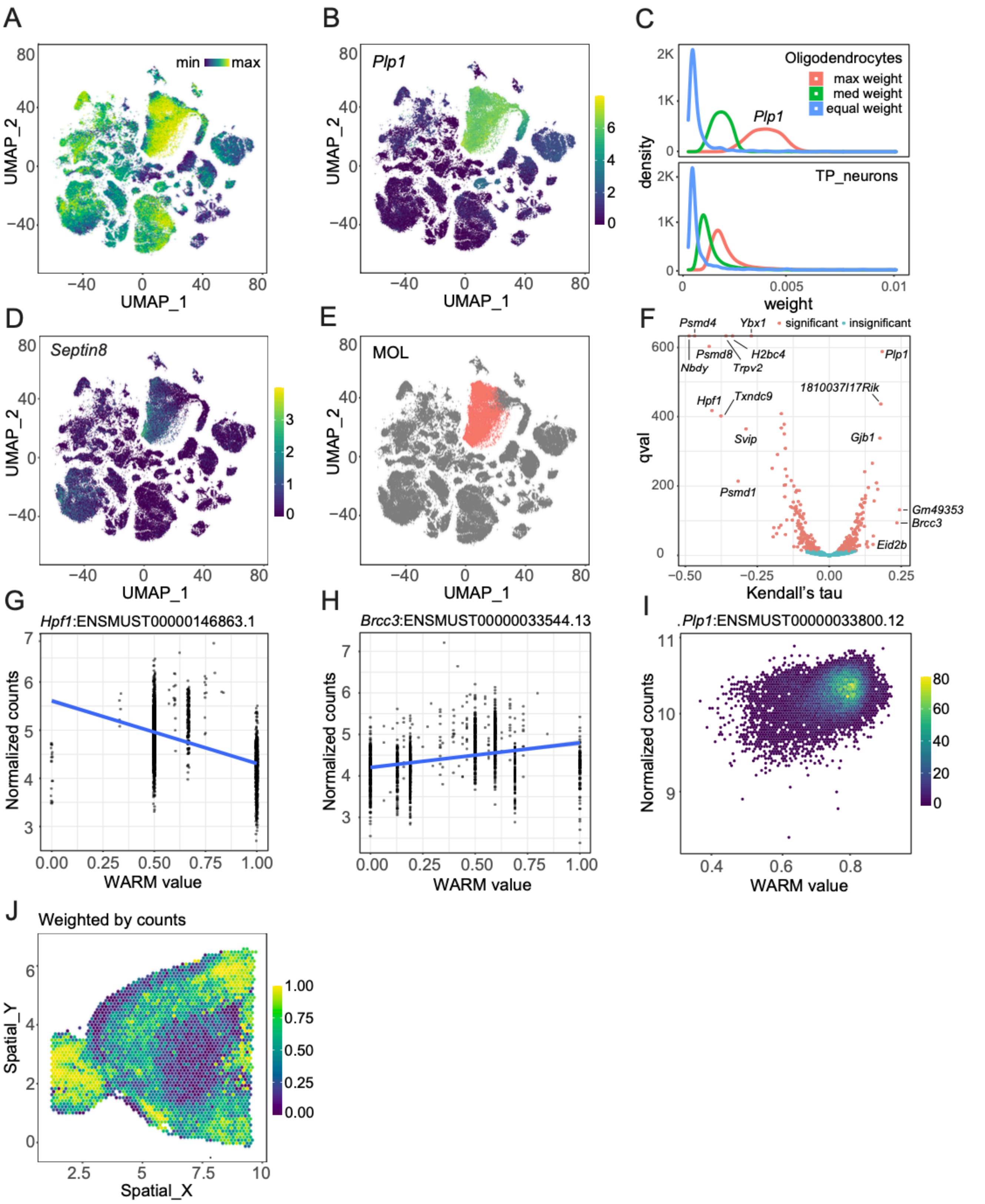

Figure S6. Cell-class-specific PAs in the adult mouse brain. Related to Figure 6.

A. UMAP showing transcriptome-wide weighted average WARM values across cells. For each cell, the WARM values over all multi-peak 3'UTRs were averaged using peak counts as weights.
B. UMAP showing *Plp1* mRNA expression.

C. Distribution of weights used in calculating transcriptome-wide average WARM for Oligodendrocytes and Telencephalon projecting neurons, respectively. Maximum, median weights are colored red and green. If treating each transcript equally, weights = 1/(number of cells with non-NA WARMs), which is shown in blue as a reference.
D. UMAP showing gene expression levels of *Septin8*.
E. UMAP highlighting Mature Oligodendrocytes (MOL).
F. Volcano plot showing the association between relative 3'UTR length and gene expression for MOL cells. The association was measured by Kendall's tau.
G. A negative linear relationship between relative *Hpf1* 3'UTR length and gene expression.
H. *Brcc3* shows a positive linear relationship between relative 3'UTR length and transcript levels.
I. Highly expressed gene *Plp1* shows a positive correlation between 3'UTR length and expression.
J. Projection of transcriptome-wide weighted average WARM values across spots onto the brain image. For each spot, the WARM values over all multi-peak 3'UTRs were averaged using peak counts as weights.

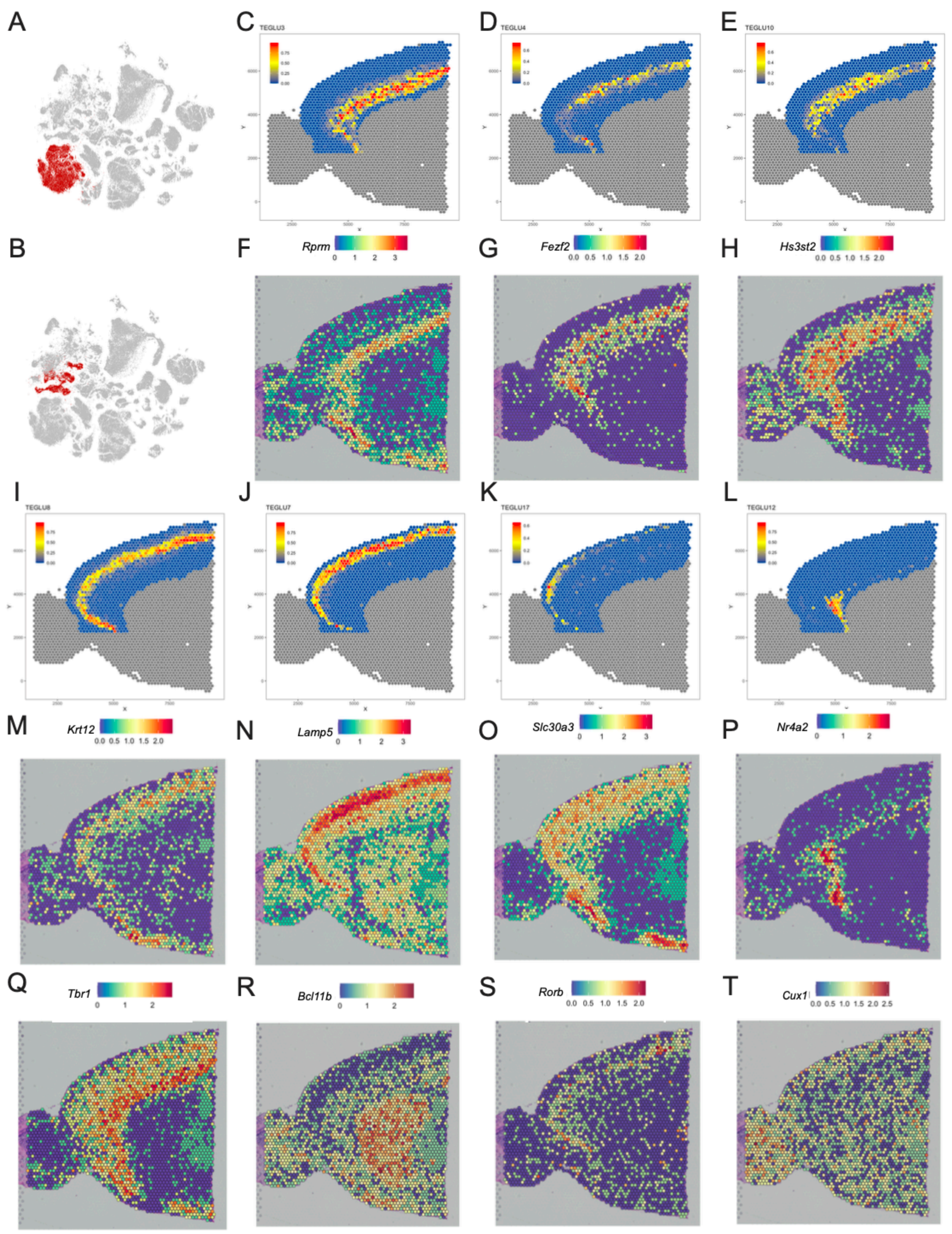

Figure S7. Cell types and marker genes in the adult mouse brain. Related to Figure 7.

A-B. Excitatory (A) and Inhibitory neurons (B) on UMAP.
C-P. Cell-type prediction probabilities (C-E, I-L) and normalized expression of marker genes (F-H, M-P) for selected telencephalon excitatory neuron (TEGLU) clusters. The prediction probabilities were calculated in Seurat when integrating scRNA-seq data into spatial transcriptomic data.
Q-T. mRNA expression of marker genes for cortical layer VI (*Tbr1*), V (*Bcl11b*), IV (*Rorb*), and II-III (*Cux1*).
